# Periodic fever, aphthous stomatitis, pharyngitis, and adenitis (PFAPA) syndrome and IgA nephropathy

**DOI:** 10.1007/s00467-012-2295-5

**Published:** 2012-09-02

**Authors:** Keisuke Sugimoto, Shinsuke Fujita, Tomoki Miyazawa, Mitsuru Okada, Tsukasa Takemura

**Affiliations:** Department of Pediatrics, Kinki University School of Medicine, 377-2 Ohno-higashi, Osaka-Sayama, Osaka 589-8511 Japan

## Abstract

**Background:**

A syndrome of periodic fever, aphthous stomatitis, pharyngitis, and adenitis (PFAPA), as well as immunoglobulin A nephropathy (IgAN), may be caused by autoimmune reactivity nephropathy.

**Case-diagnosis/treatment:**

A 10-year-old boy presented with periodic fever, exudative tonsillitis, oral aphthous ulcer, and cervical lymph node inflammation. These conditions had occurred at intervals of about 2–6 weeks since the age of 3 years. Microscopic hematuria, first detected at age 8 years, worsened during episodes of PFAPA-related fever; since 10 years of age, the hematuria was accompanied by sustained proteinuria. Examination of a kidney biopsy specimen led to a diagnosis of IgAN. In the kidney specimen, fractalkine immunoreactivity and heavy macrophage infiltration were prominent. Multi-drug cocktail therapy improved the urinalysis findings, and subsequent tonsillectomy succeeded in controlling recurrences of PFAPA and IgAN. In a post-treatment renal biopsy specimen, mesangial proliferation was decreased, and fractalkine immunoreactivity was absent.

**Conclusion:**

Immunologic reactions against certain antigens in local mucosa, including tonsils, may be impaired in PFAPA and IgAN, as evidenced by the suppression of both diseases in our patient by tonsillectomy. Accordingly, the concurrence of PFAPA and IgAN in our patient appeared to be a consequence of shared autoimmune mechanisms and systemic and local increases in cytokine concentrations, rather than coincidence.

## Introduction

Periodic fever, aphthous stomatitis, pharyngitis, and adenitis syndrome (PFAPA) is a non-genetic auto-inflammatory disease presenting in children ≤5 years of age. Although details of its pathogenesis are unclear, periodic abnormalities involving the mechanism regulating cytokine secretion have been suggested [1, 2].

Abnormalities of B and T cells have been proposed as etiologies of immunoglobulin A (IgA) nephropathy (IgAN). Increased serum IgA levels and increases in IgA-specific helper T cells or inflammatory cytokines, such as interleukin 6 (IL-6), have been implicated in the transition from IgM to IgA secretion in this disease [3, 4]. Additionally, excessive amounts of poorly galactosylated serum IgA1 appears to be the trigger for generation of glycan-specific IgG and IgA autoantibodies, resulting in the formation of circulating IgA immune complexes, which are considered pivotal to the development of nephritis [5]. Immune complexes deposited in the glomerular mesangium could promote both systemic and local secretion of various inflammatory cytokines and cell-proliferative hormones that cause a further progression of nephropathy [4].

In this report, we present a child with PFAPA and concurrent IgAN. We know of no previous report of such a case. Since a disoriented immune response triggered by various antigenic stimuli and mediated by pro-inflammatory cytokines has been suspected in both diseases, this patient is of interest in terms of the pathogenic mechanisms underlying the two diseases.

## Case presentation

**Fig. 1:**
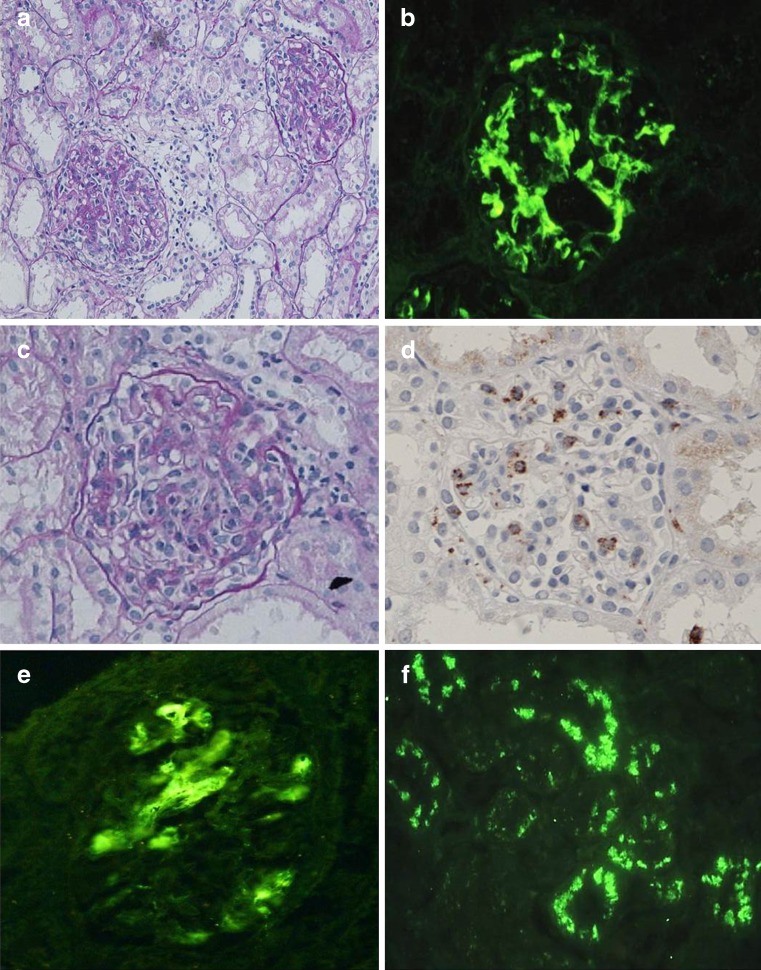
Histologic and immunohistologic evaluations of the patient’s kidney tissue. Mesangial proliferation is accompanied by crescent formation (**a**; PAS stain, ×200), and immunoglobulin A deposits are demonstrated in the mesangium by immunofluorescence (**b**; ×400). Mononuclear cell infiltration is seen in glomeruli (**c**; PAS, ×400), which is supported by immunohistologic staining (**d**; ×400). Fractalkine is demonstrated in the glomeruli (**e**; ×400) and tubular epithelium (**f**; ×200)

Since the age of 3 years, a 10 year-old boy had experienced repeated episodes of fever at or exceeding 38 °C at intervals of 2–6 weeks. In each instance, he was treated with antibiotics, but the fever persisted for several days. He was subsequently diagnosed with PFAPA. Microscopic hematuria first appeared at the age of 8 years, after which gross hematuria occurred during episodes of fever, decreasing to microscopic hematuria after the fever resolved. Sustained proteinuria appeared at the age of 10 years. Kidney biopsy, performed when the daily urinary protein excretion discharge increased to 1.0 g/day, revealed the presence of cellular crescents in addition to a diffuse, marked increase in mesangium (Fig. 1a). An immunofluorescence study of the specimen demonstrated marked granular deposition of IgA (Fig. 1b) and complement C3 deposition, primarily in the mesangium. The patient was diagnosed with IgAN. Mononuclear cell infiltration was also observed in glomeruli (Fig. 1c). Immunohistologic staining revealed glomerular infiltration of numerous CD68-positive macrophages (Fig. 1d). No amyloid deposition was detected in renal tissue. Since histologic findings suggested a poor prognosis, multi-drug cocktail therapy was initiated consisting of steroid, immunosuppressant, anticoagulant, and angiotensin-converting enzyme inhibitor (enalapril maleate), together with methylprednisolone pulse therapy. This resulted in the improvement of the urinalysis findings.

Immunohistologic staining of the pre-treatment renal tissue sample using anti-human CX3CL1/fractalkine antibody (R&D System, Minneapolis, MN) demonstrated the presence of fractalkine in glomeruli and renal tubules (Fig. 1e, f).

Based on the results of the blood tests run at the onset of nephropathy, blood urea nitrogen was 18 mg/dL, serum creatinine was 0.58 mg/dL, and creatinine clearance was 85.5 ml/min/1.48 m^2^, indicating intact renal function. Serum IgA was 275 mg/dL. During episodes of fever, increases in IL-6 (38.2 pg/mL; normal ≤4.0 pg/mL), IL-1β (24.3 pg/mL; normal ≤10 pg/mL), soluble IL-2 receptor (sIL-2) (1,240 U/mL; normal ≤500 U/mL), interferon-γ (IFN-γ; 6.3 pg/mL; normal ≤0.1 pg/mL), and soluble tumor necrosis factor (TNF) receptor-1 (2,140 pg/mL; normal ≤750 pg/mL) were observed in addition to increases in procalcitonin, erythrocyte sedimentation rate, and the white blood cell count. During intervals of alleviation of fever, IL-6 (8.7 pg/mL), IL-1β (18.5 pg/mL), and sIL-2R (784 U/mL) levels decreased; however, smaller increases persisted between episodes. Serum concentrations of various immunoglobulins, such as IgG and IgD, and of complement components were all normal. Peripheral blood CD4^+^/CD25^+^ lymphocytes, which are reported to increase in PFAPA, were reduced to 4.6 % (normal range 6–21 %). No mutation was detected in *MEFV*, a gene responsible for familial Mediterranean fever (FMF).

Tonsillectomy was performed 3 months after the cocktail therapy had been discontinued, followed by discontinuation of steroids and immunosuppressants. Both hematuria and proteinuria have subsequently remained absent for 1 year, and complete remission has been maintained. No fever or other symptom of PFAPA has occurred. On the other hand, peripheral blood CD4^+^/CD25^+^ lymphocytes remain low, although they have increased slightly to 5.5 %. In a follow-up kidney specimen obtained 1 year and 6 months after the onset of nephropathy, mesangial proliferation was decreased (Fig. 1f). Fractalkine could not be detected in either glomeruli or tubules.

## Discussion

While the exogenous or endogenous antigen(s) causing PFAPA remain unidentified, a decrease in CD4^+^/CD25^+^ lymphocytes, which maintain peripheral tolerance to the immune response and regulate natural immune responses to prevent autoimmunity, has been reported [6]. Because of consequent abnormal control of an immune response to antigenic stimulation, the levels of pro-inflammatory cytokines, such as IL-6, IFN-γ, and IL-1β, increase markedly during episodes of fever, with smaller increases persisting between episodes [7], as was observed in our patient. In PFAPA syndrome, expression of complement-related genes, such as *C1QB* and IL-1-related genes, and genes induced by IFN-γ also increase during episodes of fever [6]. Serum concentrations of T cell-derived chemokine proteins also increase in relation to febrile responses [6].

Fractalkine is produced in a form anchored to membranes, while other chemokines are in secretory form. The expression of this cytokine is enhanced by exposure to inflammatory cytokines, such as IL-1 and IFN-γ, not only in dendritic cells and nerve cells but also in renal mesangial cells and the tubular epithelium. Interaction between membrane-anchored fractalkine and fractalkine receptors (CX3CR1), which unlike other cytokines require no mediation by adhesive factors such as integrin, induces adhesion of inflammatory cells to tissues [8]. Some T cells and macrophages that infiltrate the inflammatory focus possess CX3CR1 and bind with fractalkine [8, 9]. In our study, we demonstrate intense fractalkine expression in the patient‘s glomeruli and tubular epithelium, where fractalkine may promote regional accumulation of inflammatory cells in the kidney. A recent study by Cox et al. has also demonstrated an involvement of the CX3CR1-fractalkine axis in the exacerbation of gross hematuria in patients with IgAN [10]. In addition, as a result of regional increases in the concentrations of inflammatory cytokines derived from these cells in the kidney, mesangial cell proliferation and crescent formation may be promoted, causing exacerbation of the disease. In our patient, fractalkine staining disappeared after treatment by drugs, including steroids and immunosuppressants.

Upon exposure to exogenous or endogenous antigens, circulating immune complexes containing IgA are formed; IgAN may be triggered when these are deposited in the kidney [11]. In the tonsils of IgAN patients, CD4+/CD25+ cells have been reported to be reduced, as in patients with PFAPA; local abnormalities of autoimmunity in the tonsils have also been suggested [12]. Accordingly, in our patient, we suspected that the control of immunity was disrupted by a decrease in CD4+/CD25+ cells, thereby allowing immune complexes containing IgA to be produced in excess and deposited in the kidney, with increased cytokine concentrations reflecting the infiltration of inflammatory cells into the kidney induced by fractalkine. PFAPA-associated hypercytokinemia also presumably contributed to the progression of nephropathy in our patient. Interestingly, FMF can be associated with IgAN, and the involvement of inflammatory cytokines in the onset and progression both of these diseases has been suggested [12]. While subsequent systemic immunologic mechanisms leading to the development of PFAPA and IgAN are different [5, 6], impairment of the initial immunologic tolerance against various antigens in local mucosa, including the tonsils, may be a common element of both diseases, considering that tonsillectomy is an effective treatment for not only IgAN [13] but also PFAPA, as was also the case in our patient [14]. FMF is associated with an exaggerated response to streptococcal antigens [15]. PFAPA similarly may include an inappropriate response to certain antigens impinging upon the tonsils, ultimately leading to a worsening of our patient’s urinary findings. Treatment of our patient with multi-drug cocktail therapy followed by tonsillectomy achieved satisfactory results in terms of treating both diseases. We therefore suspect that the concurrence of PFAPA and IgAN in our patient was not coincidental but rather a consequence of shared autoimmune mechanisms and systemic and local increases in cytokine concentrations.

Abnormal urinary findings, such as hematuria, have been reported in PFAPA, but not described in detail. To our knowledge, our report is the first to describe the concurrence of PFAPA and IgAN in a patient.
